# FoodEx2 Harmonization of the Food Consumption Database from the Italian IV SCAI Children’s Survey

**DOI:** 10.3390/nu16071065

**Published:** 2024-04-05

**Authors:** Laura D’Addezio, Stefania Sette, Raffaela Piccinelli, Cinzia Le Donne, Aida Turrini

**Affiliations:** 1 Council for Agricultural Research and Economics, Research Centre for Food and Nutrition, 00178 Rome, Italy; stefania.sette@crea.gov.it (S.S.); raffaela.piccinelli@crea.gov.it (R.P.); cinzia.ledonne@crea.gov.it (C.L.D.); 2 Independent Researcher, 58054 Scansano, Italy; aida.turrini@gmail.com

**Keywords:** food classification, FoodEx2, standardization of food databases, dietary surveys, food grouping, dietary pattern

## Abstract

Estimating the habitual food and nutrient intakes of a population is based on dietary assessment methods that collect detailed information on food consumption. Establishing the list of foods to be used for collecting data in dietary surveys is central to standardizing data collection. Comparing foods across different data sources is always challenging. Nomenclatures, detail, and classification into broad food groups and sub-groups can vary considerably. The use of a common system for classifying and describing foods is an important prerequisite for analyzing data from different sources. At the European level, EFSA has addressed this need through the development and maintenance of the FoodEx2 classification system. The aim of this work is to present the FoodEx2 harmonization of foods, beverages, and food supplements consumed in the IV SCAI children’s survey carried out in Italy. Classifying foods into representative food categories predefined at European level for intake and exposure assessment may lead to a loss of information. On the other hand, a major advantage is the comparability of data from different national databases. The FoodEx2 classification of the national food consumption database represented a step forward in the standardization of the data collection and registration. The large use of FoodEx2 categories at a high level of detail (core and extended terms) combined with the use of descriptors (facets) has minimized information loss and made the reference food categories at country level comparable with different food databases at national and international level.

## 1. Introduction

Estimating the habitual food and nutrient intakes of a population is based on dietary assessment methods that collect detailed information on food consumption [1]. The preparation of the list of foods and recipes to be used for data collection in dietary surveys is of central importance. It has an impact on the quantity and quality of the information collected. This is particularly the case when open-section methods are used, such as a food diary and 24 h dietary recall, which require respondents to provide detailed information about the types and amounts of food they consume [2,3]. The development of the national food list helps standardise data collection by defining food categories that are representative of the consumption of the study population. It is the core around which the dietary software tool that is used to collect and manage food consumption data is developed, facilitating interviewing and data entry, and supporting harmonized data collection. The food list is usually derived from previous surveys, includes the foods most consumed by the population, and is open-ended to allow new items to be added. It is linked to a national nutritional database to allow for energy- and nutrient-intake calculations, and to chemical occurrence data for exposure assessments.

Foods are complex objects, and thus require a dedicated information system, particularly an ontology, i.e., “a set of concepts and categories in a subject area or domain that shows their properties and the relations between them” (according to Oxford Reference [4]). The FoodOn project is the most recent example of such a construction that provides helpful information to navigate the food landscape and orienting researchers in dealing with their specific and general food-related research topics [5]. This can greatly help to develop national food lists, and helps standardize data collection and in performing total diet studies by defining food categories that are representative of the consumption of the study population [6]. In this context, the LanguaL thesaurus is a fundamental tool in which food characteristics are coded for use in categorizing single products [7,8]. Several food datasets are indexed in LanguaL, including the Food and Drug Administration (see LanguaL Indexed Datasets [9]), which has developed its own categorization system [10] that is used in national food consumption and food consumption-related studies such as NAHNES [11] and the FDA Total Diet Study [12].

In Italy, the CREA Research Centre for Food and Nutrition has conducted national dietary surveys at intervals of about 10 years since the 1980s. The most recent were conducted in 2017–2020, both on the child population and on adolescents, adults, and the elderly [13,14]. The IV SCAI children’s survey, targeting infants and children aged 3 months–9 years, led to the introduction of new items to the existing food list. In fact, the food market has been enriched over the past years by new industrially prepared food products. Health, pleasure, and convenience have been the most important and dynamic drivers of food innovation in Europe [15]. In high-income countries, there has been growing attention among consumers regarding convenience, as well as healthier, more environmentally conscious, and ethical diets [16]. To respond to changing eating habits and lifestyles, and to meet nutritional guidelines, such as reducing saturated fatty acids, free sugars, and salt, companies have generally worked to reformulate their products. The baby food market has also evolved, driven by factors such as convenience, variety, and nutritional preferences. On the product innovation front, infant food companies have increasingly focused on the quality of ingredients [17]. New branded products with special ingredients and nutritional properties, and fortified foods (infant formulae and other baby foods for complementary feeding, baby snacks and desserts, fruit and vegetable juices, breakfast cereals, and soft drinks), were introduced to the food list, as well as new dietary supplements, which also represent a continuously evolving and expanding market sector [18,19]. The characteristics of these foods must be reflected in the nomenclatures and classifications in use in a standardized way so that the information can be easily retrieved from the databases.

Comparing foods from different data sources and across countries is always challenging [20], as nomenclatures, levels of detail, and the classifications into broad food groups and sub-groups can be quite different [21]. The use of a common system for classifying and describing foods is one of the most important prerequisites for the joint analysis of data from different sources. At the European level, EFSA has addressed this need through the development and maintenance of the Food Classification and Description System for exposure assessment, FoodEx2 [22,23]. One of EFSA’s key long-term objectives is the collection of accurate and harmonized food consumption data at the European level, as set out in the Guidance on EU Menu Methodology [23], which defines the criteria for data collection, including the minimum set of food descriptors to be considered in dietary surveys (e.g., preparation/processing methods, cooking methods, preservation methods, qualitative information such as fat or sugar content, and fortification). Information on the brand and product name of manufactured and packaged foods should also be collected [24].

The aim of this work is to describe the FoodEx2 classification and description of foods, beverages, and food supplements included in the food consumption database of the IV SCAI children’s survey carried out in Italy between 2017 and 2020, as part of the EFSA EU Menu program and in compliance with the EFSA guidelines. Particular attention is given to analyzing the comprehensiveness of the classification and description system in terms of the unique codes employed versus the original food codes, and the detail level of FoodEx2 terms employed for classification. The flexibility of the classification system is also described in terms of the type and number of facets employed as additional descriptors. The information reported here could help in the interpretation and use of the consumption data from the IV SCAI children’s survey stored in the EFSA Comprehensive European Food Consumption Database (CFCD) [25].

## 2. Materials and Methods

### 2.1. Data Collection

The IV SCAI children’s survey was conducted between 2017 and 2020 by CREA Research Centre for Food and Nutrition in compliance with the Guidance on EU Menu methodology of the European Food Safety Authority (EFSA) [24]. The guidance provides recommendations for the collection of harmonized food consumption data among the EU Member States and, in particular, that dietary information should be collected for two non-consecutive days using the food diary method for infants and children and 24 h dietary recall for other age groups. Information on participants’ anthropometry, health status, physical activity, socio-economic factors, and food supplement consumption should also be collected [24]. In total, 825 children were recruited and 811 completed the survey. The reference population included all children resident in Italy aged from 3 months to 9 years at the time of the study. The studied sample was stratified by gender and age groups, namely, infants (3–11 months), toddlers (1–2 years), and other children (3–9 years). Special dietary groups or institutionalized subjects (e.g., those living in boarding schools) were not targeted for recruitment. In addition to the dietary data, a questionnaire was administered on the socio-demographic characteristics of the children and their parents/caregivers. This was used to collect information on household size, income class, education level, employment, professional category, special health conditions and special diet patterns of the child, and the child’s parents or caretakers. More details on the sample selection and survey tools can be found in [13].

Food consumption data were collected by trained field workers on two non-consecutive days using a food diary. The selection and description of foods and beverages consumed was standardized during data entry by fieldworkers using an ad hoc developed dietary software and an existing reference national list of foods, beverages, and food supplements (referred to as the “food list” in the following). The food list was largely composed of foods consumed in the INRAN-SCAI 2005-2006 food consumption survey conducted in Italy, which to date includes 3245 food items (2001 main foods, 1244 synonyms), 1523 recipes (1267 main dishes and 256 synonyms), and 439 dietary supplements. Synonyms for foods and recipes have been created to help identify the correct item during data entry, allowing for different popular and regional names [13]. New products (446 foods and beverages and 85 supplements) were added to the national food list during the IV SCAI children’s survey. All data were checked for completeness and consistency by the CREA Research Centre for Food and Nutrition team in continuous interaction with the fieldworkers. The data from the IV SCAI children’s survey are included in the CFCD [25].

### 2.2. Classification and Coding with FoodEx2

**Figure 1:**
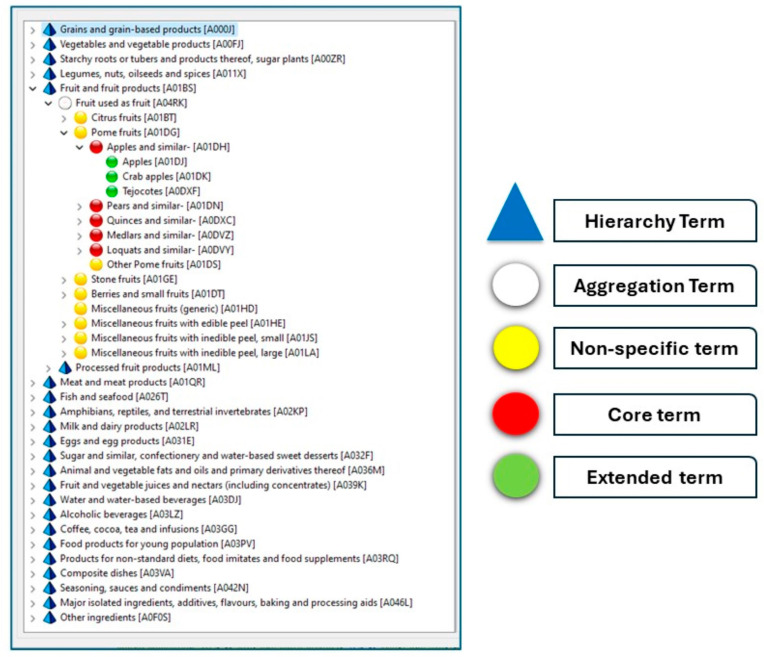
Type of FoodEx2 terms. Visualisation of the FoodEx2 tree structure, with one node expanded and display of the types of terms available for classification (available from the EFSA Catalogue Browser, Version 1.2.14 [26]).

All foods, recipes, and food supplements were classified according to the FoodEx2 system. FoodEx2 consists of a fixed and sufficiently large set of food categories defined at a high level of detail, with the “core terms” representing the minimum detail needed for intake assessment, and the “extended terms” representing more detailed categories. Core and extended terms may be aggregated in a hierarchical parent–child relationship in different ways according to the needs of the different food safety domains. Broader food categories are “hierarchy terms” (corresponding to main food groups and sub-groups and not intended to be selected for coding), “aggregation terms”, and “non-specific terms” (Figure 1). The FoodEx2 Exposure Hierarchy is the reference for the classification of food consumption databases and consists of 21 main food categories or groups that are further divided into sub-groups up to a maximum of 7 hierarchical levels in the current version [26]. At Level 1, exclusively FoodEx2 hierarchy terms (with corresponding term codes) are present (e.g., A000J—Grains and grain-based products; A01BS—Fruit and fruit products); at Level 2, almost exclusively hierarchy terms (e.g., A004V—Bread and similar products) and aggregation terms (e.g., A0BY0—Leavened bread and similar). Core and extended terms (e.g., A005F—Rye only bread and rolls; A005Y—Crackers and breadsticks) prevail at Level 3, Level 4, and Level 5. At Levels 6 and 7, exclusively or almost exclusively extended terms are present (e.g., A005H—Rye bread and rolls, wholemeal).

FoodEx2 categories (core and extended terms, and broader food categories) are also divided into three types: raw primary commodities, derivatives of raw primary commodity, and composite foods, representing three different levels of the food chain involving an increasing level of food processing. The hierarchical structure therefore organizes terms by their natural source and level of processing. The first question to be answered when classifying a food to select the most appropriate FoodEx2 base term is “What is the degree of processing”?

Another feature of FoodEx2 is that food categories (or terms) can be further detailed using additional transversal descriptors, the “facets”, therefore creating new categories that are not prefixed but respond to the requirements of the user. Facet descriptors, divided into 28 facet groups, provide additional information on food properties and attributes from various perspectives, i.e., process, production method, ingredient, qualitative information, packaging material, and target consumer. Some examples of facet groups are F01 Source, F04 Ingredient, F09 Fortification agent, F10 Qualitative-info, and F28 Process including Cooking and similar thermal preparation processes.

Whenever possible, the most precise level allowed by the system was used for the classification and coding of the items in the IV SCAI food list, namely, core or extended terms belonging to the 21 main food groups of the FoodEx2 Exposure Hierarchy.

All recipes classified under Composite dishes were disaggregated into ingredients to be incorporated in the final food consumption database, with several exceptions. According to EFSA’s instructions, composite dishes for traditional cakes, pies, biscuits, and pastries were aggregated into simple food items to be included in the EFSA Comprehensive Database and assigned a FoodEx2 code under the sub-group Fine bakery wares. Composite dishes for fruit mousses, fruit smoothies with fruit as the main ingredient, and dairy desserts were aggregated into simple foods and assigned a FoodEx2 code under in the sub-groups Processed fruit products or Fruit/vegetable juices and nectars or Dairy dessert and similar. Pizza bases were aggregated into individual foods, both for plain pizzas and for pizzas with toppings. Ingredients for egg and durum pastas, stuffed or plain, in pasta-based composite dishes were also aggregated. Foods resulting from these aggregations were given a FoodEx2 code under the sub-group Pasta, doughs and similar products. Composite dishes of sauces and gravies were also aggregated and assigned a FoodEx2 code under Condiments (including table-top formats).

The list of original food codes that was finally used for the reporting of data on food consumption in the IV SCAI children’s survey consists of 2022 items (of which 97 were food supplements/medicines containing nutrients), including synonyms, consumed as such, and variations in their consumption (e.g., adding sugar, heating, etc.) or as ingredients in recipes.

Classification and coding were performed by experts before the data collection for all the items already present in the national reference food list, and after the data collection for new items added during the survey. All data stored in the food consumption database were checked for correctness in the data transmission phase. Summary statistics were produced and possible outliers on the amounts of consumption were identified and corrected. Finally, all FoodEx2 codes were reviewed by EFSA experts during the data validation phase before being included in the CFCD. In total, 20 food groups (Level 1 of the Exposure Hierarchy) and 73 sub-groups (Level 2) were used for classification into the hierarchy tree.

## 3. Results

### 3.1. Comprehensiveness and Flexibility of FoodEx2

Table 1 shows the 20 food groups of Level 1 and, for each, the number of original codes and the number of unique FoodEx2 codes used for classification and description. A total of 1514 unique FoodEx2 codes were used, including simple and complex codes created by the coders combining basic terms with one or more descriptors. A total of 25% of the original codes were classified in Grains and grain-based products, 19% in Food product for young population, 10% in Milk and dairy products, 8% in Water and water-based beverages, 6% in Products for non-standard diets, food imitates and food supplements, 6% in Vegetables and vegetable products, and 5% in Meat and meat products. An equal proportion (4%) was classified in Fruit and vegetable juices and nectars; Sugar and similar, confectionery and Water-based sweet desserts; and Fish, seafood, amphibians, reptiles and invertebrates. A total of 3% of the original codes were classified in Fruit and fruit products, and 2% in Legumes, nuts, oilseeds and spices.

Complex codes outnumber simple codes in total and almost all main food groups. Complex codes were mostly employed for foods that required the use of base terms of the types “derivative of raw primary commodities” or “composite food”. Breads and pastas, bakery products and breakfast cereals, infant foods, fruit juices, dairy products, and food supplements, for confectionery and coffee, tea, and infusions were prevalently assigned to complex codes (Table 1). Simple codes prevailed for vegetables, fruit, legumes, nuts, oilseeds, and spices, since many items in the food list are primary commodities. Also for processed foods in these groups, there was the possibility of classification and coding with dedicated base terms (e.g., A00ZC—Preserved tomato, whole or pieces).

Table 2 focuses on food groups with a higher degree of representativeness in the food list such as cereal products, infant foods, milk and dairy products, and food supplements, since many new items were added during the survey. The number of original foods is compared to the number of unique FoodEx2 codes used, either simple or complex, for each food group (and its sub-groups).

#### 3.1.1. Grain and Grain-Based Products

A total of 392 unique FoodEx2 codes were employed to represent 505 original codes, of which 71 were simple (base term only). Simple codes were employed predominantly for breads and sweet bakery products of the food list, since the FoodEx2 categories (base terms) cover a variety of breads based on the type of flour or multigrain flours and other ingredients added. There is also an extensive list of composite food terms for biscuits, cakes, and pastries. The national food list contains many synonyms for bread, biscuits, cakes, and pastries (regional names); therefore, non-unique FoodEx2 codes were employed for FoodEx2 coding. Base terms only (simple codes) were employed for coding most cereal grains and their derivatives (e.g., A003D-Rice grain, polished; A003F-Rice flour) consumed mainly as ingredients in recipes, and only a few items were coded to add the facet for the cooking method (e.g., A003D#F28.A07GL-Rice grain, polished, PROCESS = Boiling). Simple codes were used for five breakfast cereals made from a single grain (e.g., A00DR- Rice, popped).

A total of 321 complex FoodEx2 codes were employed that were combinations of basic terms and facets. Complex codes for bakery products are mainly the result of the addition of one or more facets of the F04 Ingredient group in order to add information on special ingredients not covered by the basic term (e.g., A006F#F04.A004C$F04.A003Y$F04.A002Q$F04.A00YF—Crisp bread, wheat, refined flour, INGREDIENT = Wheat flour, durum, INGREDIENT = Wheat flour white, INGREDIENT = Maize flour, INGREDIENT = Rosemary). F22 Preparation-production place was added in many cases since packaged branded products for bread, cakes, and snacks are contained in the food list.

Breakfast cereals, which are multi-ingredient branded products often fortified with vitamins and minerals, were almost exclusively coded by complex unique codes (56 codes vs. 58 items of the food list), using facets of the groups F04 Ingredient, F09 Fortification agent (adding information on dietary components and nutrients used for fortification or enrichment of food products and food supplements), and F10 Qualitative-info (e.g., A00DR#F04.A06MF$F09.A0EVG$F09.A0EXD$F10.A0F6C—Rice, popped, INGREDIENT = Chocolate flavour, FORTIFICATION-AGENT = Vitamins, FORTIFICATION-AGENT = Iron, QUALITATIVE-INFO = Fortified).

In Pasta, doughs and similar products, apart from a few items for plain pasta, durum wheat pasta, or egg pasta, the majority had a complex code with the addition of ingredient facets for the description of filled egg pasta, or specifying the type of flour and describing pizza doughs (e.g., A008J#F28.A07GX$F04.A003Y$F04.A004C-Yeast bread—pizza dough, PROCESS = Baking, INGREDIENT = Wheat flour white, INGREDIENT = Wheat flour, durum). As a result of the aggregation of ingredients for the doughs of pizza bases, egg pasta, and durum pasta, stuffed or plain, the consumption amounts of these are included in this sub-group, while consumption of tomatoes and other pizza toppings and pasta seasonings is included in the respective groups and sub-groups.

#### 3.1.2. Food Product for the Young Population

Items for infant formulae and follow-on formulae were mostly registered with the addition of the F10.A0F6C—QUALITATIVE-INFO = Fortified facet and one or more facets from F09 Fortification agent facet group according to the information available from products’ labels (e.g., A03QL#F09.A0EXH$F09.A0EXD$F09.A0EXN$F10.A0F6C—Follow-on formula, milk-based, powder, FORTIFICATION-AGENT = Calcium, FORTIFICATION-AGENT = Iron, FORTIFICATION-AGENT = Vitamin C (ascorbic acid), QUALITATIVE-INFO = Fortified). Cereal-based foods were pasta, biscuits, and powdered infant cereals registered at the brand level. The majority were fortified foods. Therefore, facet descriptors for the ingredient used for fortification were added (F09 Fortification agent). If the information was present on the label or in the commercial name of the product, facets of the F04 Ingredient group were used to add information about the type of cereal or cereal flour used. Branded homogenized ready-to-eat meals were all coded starting with the base term according to the main ingredient (meat-based, fish-based, fruit-based, etc.) with the addition of one or more facets of the F04 Ingredient group to specify the type of meat, fish, or fruit, and other ingredients (e.g., A03RF#F28.A07LJ$F04.A01QY$F04.A01SP$F04.A00KR$F18.A07NN—Ready-to-eat meat-based meal for children, PROCESS = Homogenizing or emulsifying, INGREDIENT = Calf fresh meat, INGREDIENT = Chicken fresh meat, INGREDIENT = Leafy vegetables, PACKAGING-FORMAT = Jar).

Other foods for infants and children were mainly fruit juices and herbal infusions (based on tea or chamomile) in dry or granulated form. For both, the F04 Ingredient facets were added for the type of fruit or herb, and descriptors of the F09 Fortification facet group for fortified fruit juices.

For all baby foods that reported this information on the product label, the organic production descriptor was added (facet F21.A07SE—PRODUCTION-METHOD = Organic production).

#### 3.1.3. Milk and Dairy Products

The items for cow milk, full fat, skimmed or semi-skimmed, and human milk had simple FoodEx2 codes. Full fat or semi-skimmed facets were used for goat milk. Other items included lactose free/reduced lactose cow milk and/or cow milk fortified with addition of vitamins, minerals or probiotics (A02LY#F10.A0CQD—Cow milk, whole, QUALITATIVE-INFO = Lactose free), and fortified flavored milks (e.g., A02MP#F04.A02LZ$F04.A034H$F09.A0EXY$F09.A0EXX$F09.A0EXS$F09.A0EXL$F10.A0F6C—Flavoured milks, INGREDIENT = Cow milk, semi skimmed (half fat), INGREDIENT = Bitter-sweet chocolate, FORTIFICATION-AGENT = Vitamin B1 (thiamine), FORTIFICATION-AGENT = Vitamin B2 (riboflavin), FORTIFICATION-AGENT = Vitamin B6 (pyridoxine, pyridoxamine, pyridoxal), FORTIFICATION-AGENT = Vitamin E (tocopherols, tocotrienols), QUALITATIVE-INFO = Fortified).

Most common types of cheeses (Italian or imported) on the food list were coded univocally with simple FoodEx2 codes from the extended list (e.g., A02SS—Cheese, robiola; A02ZL—Cheese, caciocavallo). The broader core term A02SV—Firm/semi-hard cheese (gouda and edam type) was employed non-univocally for different semi-hard cheeses that are registered under different names, such as “caciotta” and similar. Complex codes were created to specify the type of milk. or more than one type, by adding the F27 Source commodity facet (e.g., A02QL#F10.A077A$F27.A02LY$F27.A02MC—Ricotta, QUALITATIVE-INFO = Full fat, SOURCE-COMMODITIES = Cow milk, whole, SOURCE-COMMODITIES = Sheep milk), or information on fat content and other quality information for processed spreadable cheeses (e.g., A031C#F10.A077C$F07.A073E—Processed cheese, spreadable, QUALITATIVE-INFO = Low fat (naturally or reduced), FAT-CONTENT = 17% fat).

A considerable number of yoghurts are included in the food list, both plain and flavored, including drinking yoghurts, which are registered under the brand name. These varied according to the type of milk (cow, sheep or goat, full fat or skimmed), % fat content, and flavor. Unique complex codes were employed, adding one or more facets for F10 Qualitative-info, to specify the type of milk (full fat or skimmed) fortification or absence of added sugar, facets from the group F09 Fortification agent for the specification of dietary components and nutrients used for fortification or enrichment, and facets from F07 Fat-content to specify the fat content as % of weight of the food item. Facets from F04 Ingredient facet group were added to specify fruit flavor or other ingredients (e.g., chocolate, cereals) for flavored yoghurts and drinking yoghurts (e.g., A02NH#F04.A06KE$F10.A077H$F10.A077K$F07.A06YJ—Yogurt, cow milk, flavoured, INGREDIENT = Apricot flavour, QUALITATIVE-INFO = Skimmed, QUALITATIVE-INFO = Without added sugar, FAT-CONTENT = 0.1% fat).

Dairy desserts are mostly ice creams registered under different brand names. Each was coded with a unique FoodEx2 code composed by a base term accompanied by the characterizing ingredient and flavor specification.

#### 3.1.4. Products for Non-Standard Diets, Food Imitations, and Food Supplements

The items classified into this group are mostly food supplements (97 products). A total of 28 products were coded as A03SL Vitamin only supplements with addition of one (Vitamin D (Cholecalciferol) or Vitamin B9 only) or more facets of F04 Ingredient group for specifying vitamins. Vitamin A (retinol, carotenoids), vitamin C, vitamins of group B, vitamin K, and vitamin E were the most commonly used facet terms. Vitamin D-only food supplements of different brands were assigned the same (non-unique) FoodEx2 codes. As these products are all in a liquid formulation, the facet term A06JL—Liquid was added to the codes (e.g., A03SL#F04.A0EXM$F03.A06JL—Vitamin only supplements, INGREDIENT = Vitamin D (cholecalciferol, ergocalciferol), PHYSICAL-STATE = Liquid).

Eight products were coded as A03SM—Mineral only supplements, with addition of one facet term to define the characterizing ingredient (fluorine, iron, or phosphorus), and one facet term for F03 Physical-state (Tablets, Fragments/granules/splinters, and mainly Liquid).

Eleven products were coded as A03SN—Combination of vitamin and mineral only supplements, with the addition of two to eighteen facet terms to define the characterizing ingredients, and one facet term for F03 Physical-state (prevalent forms were Tablets and Liquid; one product was in gum form and the stiff jelly facet was added). Vitamins of group B, vitamin A, vitamin D, vitamin D, iron, zinc, and calcium are the most common ingredients present in these products. The most common combinations are between vitamins of group B and iron, zinc, or calcium.

Eleven products were coded as A0F3Y—Probiotic or prebiotic formulations with additional facet terms employed to specify if they also contained one or more vitamin, and one facet for F03 Physical-state (prevalently F03.A06JL—Liquid).

Twelve products were coded as A03SS—Herbal formulations and plant extracts; eighteen as A03TC—Mixed supplements/formulations; three as A03SQ—Bee-produced formulations; one product as A03SX—Formulations containing special fatty acids (e.g., Omega-3, essential fatty acids); one product as A03SY—Protein and amino acids supplements; and one product as A03ST—Algae based formulations (e.g., Spirulina, chlorella). Two products that contained melatonin were assigned the non-specific code A03SV—Other common supplements, and one homeopathic medicine was assigned the non-specific code A03SP—Miscellaneous supplements or nutraceuticals.

Two dietary foods for special medical purposes were coded in the sub-group Food for particular diets (A03SF#F04.A02PR$F04.A0EXH$F04.A0EXG$F04.A0EXF$F03.A06JD—Nutritionally incomplete formulae, INGREDIENT = Milk protein, INGREDIENT = Calcium, INGREDIENT = Phosphorus, INGREDIENT = Magnesium, PHYSICAL-STATE = Powder; A03SH#F04.A0EXE$F04.A0EVR$F04.A0EVF$F10.A0B8L$F03.A06JL = Oral rehydration products, INGREDIENT = Zinc, INGREDIENT = Dietary fibre, INGREDIENT = Chemical elements, QUALITATIVE-INFO = Gluten free, PHYSICAL-STATE = Liquid).

A total of 21 products were coded as Meat and dairy imitates. Diary imitations were mainly soya, rice, or almond drinks fortified with calcium and vitamins. Two products were soyabean-based meat imitations, and one product was a frankfurter sausage imitation based on wheat protein and chickpea flour, which was assigned the broader core term A03TE—Meat imitates specifying other details through facets (A03TE#F27.A001N$F27.A013M$F28.A07LA$F28.A07JP$F28.A07JS—Meat imitates, SOURCE-COMMODITIES = Common wheat grain, SOURCE-COMMODITIES = Chickpeas (dry), PROCESS = Grinding/milling/crushing, PROCESS = Preserving with salt, PROCESS = Preserving with preserving additives).

#### 3.1.5. Other Food Groups (Fruit, Vegetables, Meat, Fish, Water, and Water-Based Beverages)

Fresh fruits were coded with simple unique FoodEx2 codes at the most detailed level of the hierarchy (extended terms). Fruits registered with different synonyms (e.g., kaki fruit or clementines) were assigned the same code. Cooked or grated fruit and fruit peel were also included for some fruits of the food list. In these cases, codes were assigned starting from the base term for fruit and adding F28 Process or F20 Part-consumed-analysed. Processed fruit products are mainly fruit purees and mousses. Unique codes were created by adding the F27 Source commodity facet and F20 Part-consumed-analysed (A01QJ#F27.A01DP$F27.A01LC$F20.A07QF—Fruit or fruit-vegetable puree, SOURCE-COMMODITIES = Pears, SOURCE-COMMODITIES = Common banana, PART-CONSUMED-ANALYSED = W/o peel). The few dried fruit products were coded by the corresponding specific FoodEx2 core terms. Entries for fruit jams are not differentiated on type of fruit, but only on sugar content (only fruit sugars or added sugars), and therefore were coded using the broader core term A01MM—Jam of fruit/vegetables and by adding details with facets (e.g., A01MM#F04.A04RK$F10.A077K—Jam of fruit/vegetables, INGREDIENT = Fruit used as fruit, QUALITATIVE-INFO = Without added sugar).

Vegetables that are usually consumed cooked are available in frozen, boiled, and raw options, the latter to be reported in the case of different cooking, multiple cooking, and/or processing occurring not changing the nature of the food, to be described case-by-case for single consumption occasions with additional facet descriptors. FoodEx2 codes therefore included simple codes for raw vegetables or were composed by adding facets of the F28 Process group (e.g., A00FR#F28.A07GL—Cauliflowers, PROCESS = Boiling). Non-unique simple FoodEx2 codes were employed for different synonyms in the food list for chicories and lettuces. Processed vegetables group included various types of processed tomatoes for which specific FoodEx2 terms, core or extended, were available. A few other items were canned or pickled products, for which either a correspondence FoodEx2 term was available (e.g., A00ZP#F20.A0F2X—Sweet corn canned, PART-CONSUMED-ANALYSED = W/o surrounding medium); otherwise, a more generic core term was employed, specifying additional information with help of facet terms for F27 Source-commodities and F06 Surrounding-medium (e.g., A0ETQ#F27.A00RS$F06.A06XK—Canned/jarred vegetables, SOURCE-COMMODITIES = Globe artichokes, SURROUNDING-MEDIUM = In olive oil).

Entries for mammalian and poultry fresh meat are included in the food list, both in cooked (usually boiled, grilled, or roasted) and raw options, the latter to be reported in case of different cooking, multiple cooking, and/or processing occurring not changing the nature of the meat, to be described case-by-case with additional facet descriptors. FoodEx2 codes therefore included simple codes for fresh meat, or were composed by adding facets of the F28 Process group (e.g., A01RJ#F28.A07GZ—Sheep (adult) fresh meat, PROCESS = Broiling/grilling). Several entries refer to a specific animal part (e.g., leg, breast); therefore, facets from F02 Part-nature were added (e.g., A01SQ#F02.A07XS—Turkey fresh meat, PART-NATURE = Breast (as part-nature)). The few entries for animal edible offal were coded with the corresponding FoodEx2 terms or by the use of a broader term then adding a facet from F02 Part-nature (e.g., A01XG—Beef liver; A01ZM#F02.A06AC—Bovine edible offal, non-muscle, other than liver and kidney, PART-NATURE = Heart (as part-nature)).

Entries for processed meat were coded with specific extended terms (e.g., A023C—Ham, beef; A024H—Italian-style sausage) of more generic core terms (e.g., A022S—Cured seasoned pork meat) or by use of F27 Source-commodities facets and other descriptors (e.g., A023X#F27.A01SP$F03.A06JA$F22.A07SH—Cooked other poultry meat, SOURCE-COMMODITIES = Chicken fresh meat, PHYSICAL-STATE = Slices, steaks or other flat cuts).

Entries for different types of fish are included in the food list in cooked (boiled, grilled/broiled), frozen, and raw/fresh options, with the latter to be selected in the case of different/multiple cooking and/or multiple processing occurring not changing the nature of the food, to be described case-by-case with additional facet descriptors. FoodEx2 codes were assigned at the most detailed level of the hierarchy (core or extended terms) corresponding to the specific common fish name, using only base terms or composed by adding facets of the F28 Process group. Entries for fish fillet were coded adding the specific facet descriptor (e.g., A02BV#F28.A07KQ$F03.A06HZ—Cod, PROCESS = Freezing, PHYSICAL-STATE = Primal cuts/fillets/halves or quarters). Non-unique simple or complex codes were assigned for synonyms of common fish names (different Italian common names for anchovies and cod).

The food list is composed of a total of 133 items for bottled mineral water registered at brand level. All these items were assigned the same simple code A03DQ—Natural mineral water. Regarding water-based non-alcoholic beverages, simple core or extended terms were used for coding cola beverages (caffeinic or non-caffeinic), bitter soft drinks (ginger ale type), and sport drinks. Complex codes were used for adding descriptors to soft drinks with fruit juice or flavor added, and diet soft drink (e.g., A03FY#F10.A077L—Diet soft drink with caffeine, QUALITATIVE-INFO = Sugar free).

### 3.2. Detail Level of FoodEx2 Classification

Based on the characteristics of the base terms, the distribution of the FoodEx2 codes employed was analyzed across the seven levels of the hierarchy and by the level of detail of the terms (Table 3). A total of 1514 unique FoodEx2 codes were used or created ad hoc. These include codes which are formed from the same base term but are differentiated by facet terms (e.g., A03RA—Biscuits, rusks and cookies for children; A03RA#F04.A001X$F10.A0F6C—Biscuits, rusks and cookies for children, INGREDIENT = Mixture of grains, QUALITATIVE-INFO = Fortified).

A total of 22% of the codes are at Level 3. Most of the codes are at Level 4 (33%) and Level 5 (34%), 10% are at Level 6, and one code at Level 7 (representing 0.06%) was used for Tiramisù (A00AS#F04.A009X$F04.A02QH$F04.A03KC$F04.A03HG—Cream cheese cake, INGREDIENT = Biscuits, sweet, plain, INGREDIENT = Mascarpone, INGREDIENT = Coffee (average strength) beverage, INGREDIENT = Cocoa powder). In almost all food groups, 80% or more of the codes are concentrated at Levels 4 and 5 of the hierarchy. For Milk and dairy products, the used codes were concentrated at Levels 4 (46%) and 6 (38%). In Food products for young population, the codes were concentrated at Level 3 (84%), with the remaining at Levels 5 (14%) and 2 (1%).

About 90% or more of the FoodEx2 codes used started from the basic terms at the maximum detail in the hierarchy structure (core terms or extended terms). Several aggregation terms (broader categories) were employed for coding cakes (Cereal and cereal products group), for legumes, milk, and sugars.

## 4. Discussion

The development of food databases requires the identification of foods through an appropriate nomenclature and accurate description. Food nomenclatures and descriptive systems may use different levels of detail depending on the area of interest and application. The terminologies used in the different sectors are often not compatible. This makes it difficult to exchange data between countries, between scientific disciplines, or between organizations within the same country. A consistent food description system is essential when comparing and exchanging data across databases [27]. The level of detail required to describe and identify a food generally increases with the number of different processes and treatments. Foods “as consumed” require many attributes for accurate description, and more detail is needed when dealing with composite foods (combining two or more individual processed foods) [28]. Ideally, food description should be highly detailed to allow aggregation of foods with similar characteristics from different perspectives. On the other hand, the information varies depending on the type of food and different areas of interest and a detailed description requires a high expertise.

The FoodEx2 system represent a compromise between the above needs: possibility of descriptive detail, applicability across multiple domains, and the need for aggregation. It uses pre-defined classification categories (“base terms”) that already contain some implicit characteristics. This limits the use of additional descriptors (facets) to those cases where they are needed, thereby limiting the number of errors, and saving time. The FoodEx2 system was developed to support the collection of accurate and comparable data across different food and feed safety domains. It has been designed to be generally applicable and to have the ability to link all the different food databases. Launched in 2011, FoodEx2 has been revised and improved following an intensive testing period for the collection of food consumption and chemical occurrence data. The current version (revision 2) was released in 2015 [22]. The system is regularly maintained to keep the terminology up to date with scientific and legislative requirements. To date, six major updates have been carried out, including the addition of new categories (new terms), corrections to existing ones, the withdrawal of terms, and changes to hierarchical relationships [23].

Currently, FoodEx2 harmonized food consumption data from 16 child and 20 adult surveys that have been conducted in most European countries as part of the EU Menu data collection are stored in the EFSA CFCD [25]. The database described here is part of the CFCD. Summary statistics on chronic and acute food consumption, for different age groups and most European countries, are available from the EFSA’s website. The Comprehensive Database is currently used for estimation of exposure to different substances (biological hazards, contaminants, food additives, novel foods, and nutrients, to name but a few); see, for example, [29]. EFSA has developed several tools to estimate dietary exposure to food-borne chemical hazards, e.g., the Food Additives Intake Model (FAIM) and the Dietary Exposure tool (DietEx), which are based on FoodEx2 and using individual level food consumption data at the lowest level of granularity from the CFCD data.

FoodEx2 is currently used at a global level with the support of the Food and Agriculture Organization (FAO) of the United Nations and the World Health Organization (WHO) to harmonize datasets in the FAO/WHO Global Individual Food consumption data Tool—FAO/WHO GIFT [30]. To date, the inventory map contains harmonized and shared information on 53 surveys, 26 datasets in preparation for sharing, and 241 surveys identified as suitable for sharing, for a total of more than 100 countries in the world [31].

In 2018, the European Food Safety Authority (EFSA) established an official collaboration for harmonizing individual-level dietary data using the FoodEx2 food classification and description system with the Global Dietary Database (GDD) [32]. GDD aims to perform novel research and translation on global dietary intakes, diet-related disease burdens, and evidence-based policy actions to create a healthier, equitable, and more sustainable food supply [33,34].

The PO2/TransformON, an ontology on food, feed, bioproducts, and biowaste engineering for data integration in a circular bioeconomy and nexus-oriented approach, is also based on FoodEx2 for the food and feed hierarchies. This ontology addresses animal and plant food sources, food categories, and products [35], and the FoodEx2′s approach is well suited to the strategy of following a process of transformation, as the classification is based on the identification of the most relevant treatments to create new natures of products from the raw materials and on the creation of specific food groups for the derivatives obtained with these treatments.

Moreover, in 2016, some authors developed and tested the feasibility of a method for establishing harmonized Total Diet Study (TDS) food and sample lists in five European countries with different consumption patterns. Since the national data from these five countries were not comparable, all foods were linked to the EFSA FoodEx2 classification and description system [36]. Total Diet Studies (TDSs) have been used as a tool for estimating the level of dietary exposure to chemical substances among the general population since the 1960s [37].

In addition to the IV SCAI children’s survey and the IV SCAI survey on adolescents, adults, and the elderly, food consumption data from the INRAN SCAI 2005-2006 survey on the whole Italian population (0–97 years) are also mapped in FoodEx2 and are available through the EFSA’s CFCD and the FAO/WHO GIFT platform [31]. FoodEx2 mapping represented an important step forward in standardizing the information related to the food consumed by the Italian population and in the harmonization and comparability of the national dietary data at the European and global level. Additionally, data from the Italian Nutrition & Health Survey (INHES), conducted in 2010-13 on the adult population (>=18 years), are mapped in FoodEx2 [38]. However, there are several major differences in the way consumption data are reported in the Comprehensive Database between the present and the past INRAN-SCAI 2005-06 survey. As detailed in Methods, composite dishes for traditional cakes, pies, biscuits, and pastries were aggregated and assigned a FoodEx2 code of the sub-group Fine bakery wares. Doughs of pizza bases in composite dishes for pizza, and ingredients of egg pasta and durum pasta in pasta-based composite dishes, were aggregated into individual foods and assigned a FoodEx2 code of the sub-group Pasta, doughs and similar products. In the previous survey, these composite dishes were disaggregated for inclusion in the database; therefore, the consumption amount of single ingredients such as flour, egg, and sugar was registered in the respective food sub-groups (Cereals and cereal primary derivatives; Eggs and egg products; Sugar and similar, confectionery and water-based sweet desserts). These differences must be taken into account, particularly when comparing the consumption of foods falling under the named sub-groups (cakes, biscuits, pastries, pasta, pizza, flour, eggs, and sugar) between the previous and the current survey.

The dietary software tool used in the survey was structured to record many details of an individual’s food consumption. It allowed the selection of personalized facet descriptors (e.g., cooking method and other processing facets) through drop-down menus corresponding to each food item selected from the database and to the ingredients of composite dishes. A personalized food description could either match that already included in the selected original code, and therefore be assigned the FoodEx2 code, or modify/add to the original description (e.g., for “codfish breaded and fried” the original food code selected was “Fish, codfish, whole, raw” (FoodEx2 code A02BV—Cod), personalized facets added were F28.A07HK-PROCESS—Breading and F28.A07GV-PROCESS—Deep frying, leading to modified code A02BV#F28.A07HK$F28.A07GV-Cod, PROCESS = Breading, PROCESS = Deep frying for the individual consumption). This additional information, mainly culinary preparation and cooking facets, recorded at an individual level and for each consumption occasion, is beyond the scope of this work. It was recorded during the survey and stored in the database for restricted internal use. It can be used to refine the nutrient intake estimation and for other specific purposes. For composite dishes, the diet software allowed the recording of personalized recipes, allowing for the removal/addition of ingredients that did not change the main characteristics of the recipe, as well as the addition of personalized facets to the ingredients. However, because of the aggregation of several composite dishes, as described in Methods, a unique food item code replaced all personalized recipes of the same composite dish. This resulted in a loss of detail of information linked to nutrient content of specific ingredients of recipes.

Having combined different age groups of children (e.g., including infants) has influenced the type of foods included in the database. After cereal products, baby foods are the second most represented food group. This is also reflected in the representation of food consumption in the 3–11 months age group, as foods for the young population are among the most consumed, both in terms of quantity and percentage of consumers, whereas in the 3–9 years age group, they are hardly consumed. Indexing these foods represented a thorough test of FoodEx2 application and, at the same time, an opportunity to enrich the consumption database in terms of standardized information.

The kind of information provided by the present work can be very helpful for the completion of thesauri of food items. This is necessary because the FoodEx2 (and the former FoodEx1) gathers principles from previous coding systems prepared at the international level, and particularly the Eurocode approach with variable fields [7] but considering aggregation levels crucial for the needs of building variables to statistically analyze dietary patterns and make suitable the interpretation of diet quality with a reasonable number of food categories [18,28].

Describing foods is crucial to appropriately aggregate food items and to link both food consumption data and food composition-related data [28], and food consumption data and occurrences of food contaminants data like in the MCRA system [39]. To make this possible, food descriptions, like the LanguaL system approach where all food characteristics are coded [7], and naming of components, like the INFOODS [40] and EuroFIR [41] approaches, are required. Food classification is necessary to describe dietary patterns in a synthetic way for the users, facilitating the assignment of food composition to food consumption data [42] for researchers, warranting the comparability [27]. Food description is the first step to create a synthetic code to use in food data processing, and the LanguaL system provides descriptors to standardize a complete food description [7]. The second step is to aggregate foods, and the facet A in LanguaL provides the full mapping to international food classification systems, including a full mapping to FoodEx2 [8].

The IV SCAI individual food consumption data followed the EU Menu approach [24], representing an elaboration of a long experience in the food classification work from the Eurocode project to the EuroFIR approach and further [43].

This work focuses on the comprehensiveness of FoodEx2 as the ability to assign as many unique codes as possible corresponding to the original food codes, thus minimizing the loss of information. On the one hand, it depends on the number of base terms available for classification, which is sufficiently large though not exhaustive. On the other hand, it is closely linked to the flexibility of the system, represented by the use of facets for further characterization of the selected food category (or code). The type and number of descriptors added can vary greatly and do not only depend on the food to be described. Most FoodEx2 categories are generic. This allows the user to classify several foods under one category and differentiate them with facets. Other categories are specific and do not require further description. Although base rules for classification and coding are established [22], the descriptive part of the codes relies heavily on the knowledge and discretion of the coder.

All food databases are built starting from the available items collected in population studies or available food item databases, and this represents a major strength and weakness to be tackled, at the same time. The possibility to add new items is the strength, in addition to the acquisition of new core terms and new facets. Given the rapid evolution of the food market, a continuous updating of the catalogues underpinning the food codification (see, e.g., [44]) is required. In this regard, standard procedures to update the system need to be implemented [45]. The current EFSA’s catalogue does not include some core terms (e.g., goose eggs) and some facets (e.g., drying, sparkling), so requests must be sent to the authority technical group. Organized data systems adopt procedures to update the information, either from contributors or users maintaining the database structure.

One main strength of the FoodEx2 indexed database is being part of the EU Menu food consumption data collection, which uses a harmonized methodology providing comparable information across most countries and regions in the EU. Moreover, one of the undoubted advantages of this classification system is that it is able to address the challenge of differences in food classifications and descriptions [34], ensuring that foods from different surveys are described and classified in the same way by applying standard procedures to the datasets in order to have them in the same format, despite the fact that national food consumption data are not fully comparable [30,36]. This is certainly the case for the classification aspect of the system. In terms of its application as a descriptive system, the flexibility in the use of descriptors is a strong point. This has allowed many unique codes to be created for food products. Another advantage in terms of coding time is the use of only the necessary descriptors. However, this also introduces an element of discretion and a lack of systematicity on the part of the coder regarding the type and number of descriptors that are used.

To the best of our knowledge, this is the first work reporting a detailed description of the FoodEx2 mapping of individual foods included in the consumption database of a national dietary survey in Italy. Other experiences of FoodEx2 harmonization included, for example, the items stored in a Dietary Supplement Label Database developed according to products’ availability in the Italian market and including items consumed in Italian dietary surveys [18].

Although food consumption data at an individual level and FoodEx2 codes complete with all facet descriptors have restricted use, summary statistics from the database are publicly available on EFSA’s website, by age classes and FoodEx2 food groups, from Level 1 to Level 7. In this regard, the information here presented could support the exploration of summary statistics and their interpretation. For example, in Table 3, codes for *Legumes* are all from Level 5 of the hierarchy (100% of codes). This means that summary data can be obtained for broader food groups (e.g., at Levels 1 and 2) and for more detailed groups up to Level 5 if the number of consumers allows robust estimates. However, no consumption data are available for food categories at Level 6 and Level 7.

## 5. Conclusions

The present work reports on the experience of using the FoodEx2 system applied to a food consumption database, providing information on the database structure and an example of database standardization and enrichment by the type and number of structured pieces of information (facets) added. The information reported here could also support the interpretation and use of the consumption data from the IV SCAI children’s survey, publicly available through the CFCD, in addition to the advantage of European and global comparability of the adopted FoodEx2 classification. Although this type of exercise is very laborious, it is recommended when managing the complex and diverse information embedded in food databases to facilitate the retrieval of information in several specific domains, thus improving and facilitating data processing. However, further efforts are needed to make the collection and storage of food information increasingly systematic and standardized.

## Figures and Tables

**Table 1:** Number of original national food list food codes and number of unique, simple and complex FoodEx2 codes used by food group at Level 1.

Food Group (Level 1—Exposure Hierarchy)	Original Codes (n)	FoodEx2 Unique Codes (n)	Simple ^1^ FoodEx2 Codes (n)	Complex ^2^ FoodEx2 Codes (n)
Grains and grain-based products	505	392	71	321 (1–16)
Food products for young population	382	277	5	272 (1–20)
Milk and dairy products	197	160	28	132 (1–13)
Water and water-based beverages	156	17	9	8 (1–3)
Products for non-standard diets, food imitates and food supplements	120	105	6	99 (1–19)
Vegetables and vegetable products	118	97	49	48 (1–7)
Meat and meat products	96	81	18	63 (1–3)
Fruit and vegetable juices and nectars (including concentrates)	87	68	8	60 (1–10)
Sugar and similar, confectionery and water-based sweet desserts	72	58	13	45 (1–6)
Fish, seafood, amphibians, reptiles and invertebrates	71	62	28	34 (1–2)
Fruit and fruit products	67	55	29	26 (1–4)
Legumes, nuts, oilseeds and spices	48	45	25	20 (1–4)
Coffee, cocoa, tea and infusions	32	31	1	30 (1–10)
Seasoning, sauces and condiments	24	23	15	8 (1–3)
Animal and vegetable fats and oils and primary derivatives thereof	17	13	9	4 (1–2)
Starchy roots or tubers and products thereof, sugar plants	13	13	4	9 (1–7)
Eggs and egg products	7	7	6	1 (1–1)
Major isolated ingredients, additives, flavours, baking and processing aids	5	5	3	2 (1–3)
Alcoholic beverages	4	4	2	2 (1)
Other ingredients	1	1	1	-
**TOTAL**	**2022**	**1514**	**331**	**1183**

^1^ FoodEx2 codes composed by a base term only. ^2^ FoodEx2 codes composed by a base term and at least one facet descriptor. Minimum and maximum number of facet descriptors used to form complex FoodEx2 codes are reported in parentheses.

**Table 2:** Number of original national food list codes of most representative food groups, and number of unique, simple, and complex FoodEx2 codes used by group (Level 1) and sub-group (Level 2).

Food Group (Level 1) and Sub-Group (Level 2)	Original Codes (n)	FoodEx2 Unique Codes (n)	Simple ^1^ FoodEx2 Codes (n)	Complex ^2^ FoodEx2 Codes (n)
**Grains and grain-based products**	**505**	**392**	**71**	**321**
Cereals and cereal primary derivatives	27	25	21	4
Bread and similar products	123	83	15	68
Pasta, doughs and similar products	56	47	6	41
Fine bakery wares	241	181	24	157
Breakfast cereals	58	56	5	51
**Food product for young population**	**382**	**277**	**5**	**272**
Infant and follow-on formulae	53	40	4	36
Other food for infants and children	21	16	0	16
Processed cereal-based food for infants and young children	108	60	1	59
Ready-to-eat meal for infants and young children	200	161	0	161
**Products for non-standard diets, food imitates and food supplements**	**120**	**105**	**6**	**99**
Food for particular diets	2	2	0	2
Food supplements and similar preparations	97	82	0	82
Meat and dairy imitates	21	21	6	15
**Milk and dairy products**	**197**	**160**	**28**	**132**
Cheese	77	58	22	36
Dairy dessert and similar	34	29	0	29
Fermented milk or cream	46	40	1	39
Milk and dairy powders and concentrates	2	2	2	0
Milk, whey and cream	38	31	3	28

^1^ FoodEx2 codes composed by a base term only. ^2^ FoodEx2 codes composed by a base term and at least one facet descriptor.

**Table 3:** Number of unique FoodEx2 codes used to classify the national food list, their distribution (n) in main FoodEx2 groups (Level 1) and sub-groups (Level 2), and their distribution (%) by Exposure Hierarchy level and hierarchy term detail.

Food Group (Level 1) and Sub-Group (Level 2)	FoodEx2 Unique Codes (n)	Level 2 ^1^ (%)	Level 3 ^1^ (%)	Level 4 ^1^ (%)	Level 5 ^1^ (%)	Level 6 ^1^ (%)	Level 7 ^1^ (%)		M ^2^ (%)	P ^2^ (%)	C ^2^ (%)	E ^2^ (%)
**Grains and grain-based products**	**392**	**-**	**8**	**35**	**44**	**13**	**0.3**		**5**	**1**	**48**	**47**
Cereals and cereal primary derivatives	25	-	-	24	44	32	-		-	-	24	76
Bread and similar products	83	-	23	40	34	4	-		4	1	71	24
Pasta, doughs and similar products	47	-	-	11	45	45	-		9	2	23	66
Fine bakery wares	181	-	7	44	40	8	1		6	-	40	55
Breakfast cereals	56	-	-	25	71	4	-		4	-	71	25
**Vegetables and vegetable products**	**97**	**-**	**5**	**35**	**47**	**12**	**-**		**1**	**-**	**9**	**90**
**Starchy roots or tubers and products thereof, sugar plants**	**13**	**-**	**-**	**69**	**31**	**-**	**-**		**-**	**-**	**-**	**100**
**Legumes, nuts, oilseeds and spices**	**45**	**-**	**9**	**11**	**73**	**7**	**-**		**11**	**-**	**9**	**80**
Legumes	16	-	-	-	100	-	-		-	-	-	100
Nuts, oilseeds and oilfruits	14	-	14	-	79	7	-		21	-	-	79
Spices	10	-	20	20	40	20	-		20	-	20	60
Processed legumes, nuts, oilseeds and spices	5	-	-	60	40	-	-		-	-	40	60
**Fruit and fruit products**	**55**	**-**	**2**	**33**	**55**	**11**	**-**		**-**	**2**	**91**	**7**
Fruit used as fruit	33	-	3	-	88	9	-		-	3	97	-
Processed fruit products	22	-	-	82	5	14	-		-	-	82	18
**Meat and meat products**	**81**	**-**	**1**	**22**	**65**	**11**	**-**		**-**	**1**	**35**	**64**
**Fish, seafood, amphibians, reptiles and invertebrates**	**62**	**-**	**-**	**10**	**77**	**13**	**-**		**-**	**2**	**66**	**32**
**Milk and dairy products**	**160**	**-**	**7**	**46**	**9**	**38**	**-**		**4**	**3**	**44**	**49**
Cheese	58	-	7	47	16	31	-		-	-	43	57
Dairy dessert and similar	29	-	24	76	-	-	-		24	-	76	-
Fermented milk or cream	40	-	-	28	8	65	-		-	10	25	65
Milk and dairy powders and concentrates	2	-	-	-	100	-	-		-	-	-	100
Milk, whey and cream	31	-	-	42	3	55	-		-	-	45	55
**Eggs and egg products**	**7**	**-**	**-**	**71**	**29**	**-**	**-**		**-**	**-**	**100**	**-**
**Sugar and similar, confectionery and water-based sweet desserts**	**58**	**-**	**16**	**36**	**43**	**5**	**-**		**10**	**9**	**74**	**7**
**Animal and vegetable fats and oils and primary derivatives thereof**	**13**	**-**	**15**	**23**	**62**	**-**	**-**		**-**	**8**	**62**	**31**
**Fruit and vegetable juices and nectars (including concentrates)**	**68**	**-**	**15**	**84**	**1**	**-**	**-**		**-**	**15**	**84**	**1**
**Water and water-based beverages**	**17**	**-**	**-**	**53**	**24**	**23**	**-**		**-**	**-**	**65**	**35**
Drinking water	5	-	-	100	-	-	-		-	-	100	-
Water based beverages	9	-	-	11	44	44	-		-	-	33	67
Beverages concentrates	3	-	-	100	-	-	-		-	-	100	-
**Coffee, cocoa, tea and infusions**	**31**	**-**	**16**	**68**	**16**	**-**	**-**		**3**	**6**	**35**	**55**
**Alcoholic beverages**	**4**	**-**	**50**	**50**	**-**	**-**	**-**		**-**	**-**	**50**	**50**
**Food products for young population**	**277**	**1**	**85**	**-**	**14**	**-**	**-**		**1**	**-**	**85**	**14**
Infant and follow-on formulae	40	-	-	-	100	-	-		-	-	-	100
Other food for infants and children	16	12	88	-	-	-	-		12	-	88	-
Processed cereal-based food for infants and young children	60	-	100	-	-	-	-		-	-	100	-
Ready-to-eat meal for infants and young children	161	-	100	-	-	-	-		-	-	100	-
**Products for non-standard diets, food imitates and food supplements**	**105**	**-**	**19**	**65**	**16**	**-**	**-**		**-**	**2**	**86**	**12**
Food for particular diets	2	-	-	100	-	-	-		-	-	100	-
Food supplements and similar preparations	82	-	23	77	-	-	-		-	2	98	-
Meat and dairy imitates	21	-	5	14	81	-	-		-	-	38	62
**Seasoning, sauces and condiments**	**23**	**-**	**13**	**43**	**39**	**4**	**-**		**-**	**-**	**70**	**30**
**Major isolated ingredients, additives, flavours, baking and processing aids**	**5**	**-**	**40**	**40**	**20**	**-**	**-**		**-**	**-**	**60**	**40**
**Other ingredients**	**1**	**-**	**100**	**-**	**-**	**-**	**-**		**-**	**-**	**100**	**-**
**TOTAL**	**1514**	**0.1**	**22**	**33**	**34**	**10**	**0.06**		**3**	**2**	**58**	**37**

^1^ Levels up to a maximum of 7 into which the 20 food groups (Level 1) (listed in bold character in the header column) are further divided in the FoodEx2 hierarchical tree. ^2^ M = Aggregation terms; P = Non-specific terms; C = Core terms; E = Extended terms.

## Data Availability

The original contributions presented in the study are included in the article, further inquiries can be directed to the corresponding author.
